# Malignant Mesenteric Perivascular Epithelioid Cell Neoplasm Presenting as an Intra-Abdominal Fistula in a 49-Year-Old Female

**DOI:** 10.1155/2014/534175

**Published:** 2014-07-10

**Authors:** Sakshi Kapur, Napoleon K. Patel, Miles B. Levin, Richard Huang

**Affiliations:** ^1^Department of Internal Medicine, Overlook Medical Center, 99 Beauvoir Avenue, Summit, NJ 07902, USA; ^2^Division of Pathology, Overlook Medical Center, 99 Beauvoir Avenue, Summit, NJ 07902, USA; ^3^MS III, St. George's University School of Medicine, True Blue, Grenada

## Abstract

Perivascular epithelioid cell tumors are rare mesenchymal tumors arising from histologically and immunohistochemically distinctive perivascular epithelioid cells that express both myogenic and melanocytic markers. These tumors are known to arise from different organs in the body and usually have an unpredictable clinical course. We report a case of a 49-year-old female who presented with diffuse abdominal pain, fever, chills, and nonbilious vomiting for a day. Work-up revealed a mesenteric mass measuring 13.5 × 7.7 × 9.5 cm, arising in the mesentery of the hepatic flexure, with adjacent gas suggestive of fistularization into the right colon. An exploratory laparotomy with resection of the mesenteric mass was performed, and the initial histopathology results were compatible with either an adenocarcinoma or a sarcoma; however, because of poor differentiation it was difficult to make a definitive diagnosis. However, final histopathology results revealed a malignant perivascular epithelioid cell tumor (with reservation that a S100 negative metastatic melanoma must be excluded clinically). Following surgery the patient was started on everolimus, an m-TOR inhibitor, and has shown good response to this medication.

## 1. Introduction

Perivascular epithelioid cell tumors (PEComas) are rare mesenchymal tumors, expressing both myogenic and melanocytic markers and lacking a normal cellular counterpart. PEComas are known to arise from different organs such as the kidneys, lungs, liver, pancreas, prostate, and female genital tract. They are also associated with tuberous sclerosis complex. Different pathologies include angiomyolipomas, lymphangioleiomyomatosis, clear-cell “sugar” tumor, and clear-cell myomelanocytic tumor. Malignant PEComas are rare and very few cases have been reported in literature. Surgery remains the cornerstone of treatment, although treatment modalities are still controversial, especially in advanced conditions.

## 2. Case Report

A 49-year-old female with no significant past medical or surgical history presented to our hospital with sudden onset of diffuse abdominal pain for four hours. She reported fever, chills, nausea, and nonbilious vomiting for one day. Two weeks previously, she was diagnosed with a mass arising in the mesentery of her right colon and was scheduled for an elective surgery. Both computed tomographies of her chest and colonoscopy were unremarkable. However, the day prior to her surgery she developed the above symptoms and was hospitalized.

Physical examination revealed an average sized female in acute distress secondary to abdominal pain. Vitals signs were as follows: temperature: 102.4 F, blood pressure: 128/74 mm of Hg, and pulse: 120 beats per minute and respiratory rate of 16 per minute. The abdomen was diffusely tender on palpation and positive guarding and rigidity, and no hepatosplenomegaly was noted. Both heart and lungs were normal on exam.

Work-up revealed the following: hemoglobin 8.6 g/dL (normal range: 12.5–16.0 g/dL), white blood cell count 12.1/nL (normal range: 4.5–11.0/nL), and platelet count 322/nL (normal range: 150–450/nL). Blood urea nitrogen, serum creatinine, and electrolytes were within normal limits. Both serum amylase and lipase were within reference range. Total bilirubin was 0.9 mg/dL (normal range: 0.0–1.1 mg/dL), aspartate aminotransferase (AST) 74 U/L (normal range: 15–37 U/L), alanine aminotransferase (ALT) 57 U/L (normal range: 12–78 U/L), and alkaline phosphatase 432 U/L (normal range: 50–136 U/L).

**Figure 1:**
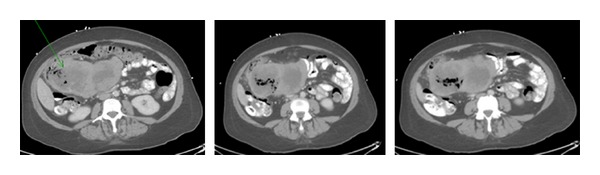
Computed tomography of the abdomen showing a retroperitoneal mesenteric mass with fistulization (communication) into the right colon.

**Figure 2:**
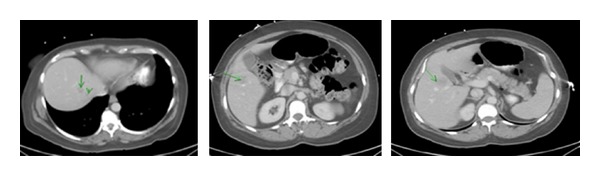
Computed tomography of the abdomen showing multiple metastatic lesions in various segments of the liver.

Computed tomography of the abdomen revealed a large mass measuring approximately 13.5 × 7.7 × 9.5 cm arising from the mesentery of the hepatic flexure, with central necrosis and mixed attenuation most likely secondary to intratumoral abscess, with gas adjacent to this mass suggestive of fistulization (communication) with the bowel. Multiple low attenuation hepatic lesions were identified, such as in the anterior segment of the right lobe measuring 2.3 cm, in the lateral segment of the left lobe measuring 1.9 cm, and near the dome of the posterior segment of the right lobe measuring 1.3 cm. No free air, ascites, or frank evidence for lymphadenopathy was noted. The spleen, pancreas, gallbladder, adrenal glands, and kidneys were unremarkable (Figures 1 and 2).

**Figure 3:**
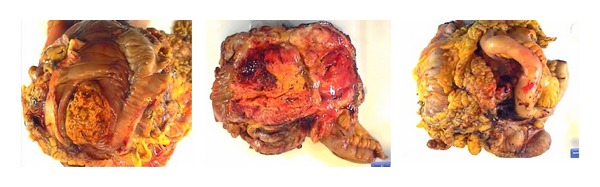
Surgical specimen showing resected mesenteric mass, fistularizing into the colon and right hemicolectomy.

Patient was started on intravenous fluids and antibiotics. Abdominal exploration (laparotomy) with resection of the mesenteric mass was performed. Frozen section results were compatible with either an adenocarcinoma or a sarcoma; however, because of poor differentiation, it was difficult to make a definitive diagnosis. Obvious metastases to the liver and peritoneum were also noted. Since the mass had fistulized into the right colon, a palliative right hemicolectomy was performed (Figure 3). The retroperitoneal mass was also noted to invade the transverse colon at two different sites of small bowel. Both the transverse colon and small bowel were resected at suitable sites. A side-to-side functional end-to-end ileocolostomy was performed. Patient tolerated the procedure well and her postoperative period was uneventful.

**Figure 4:**
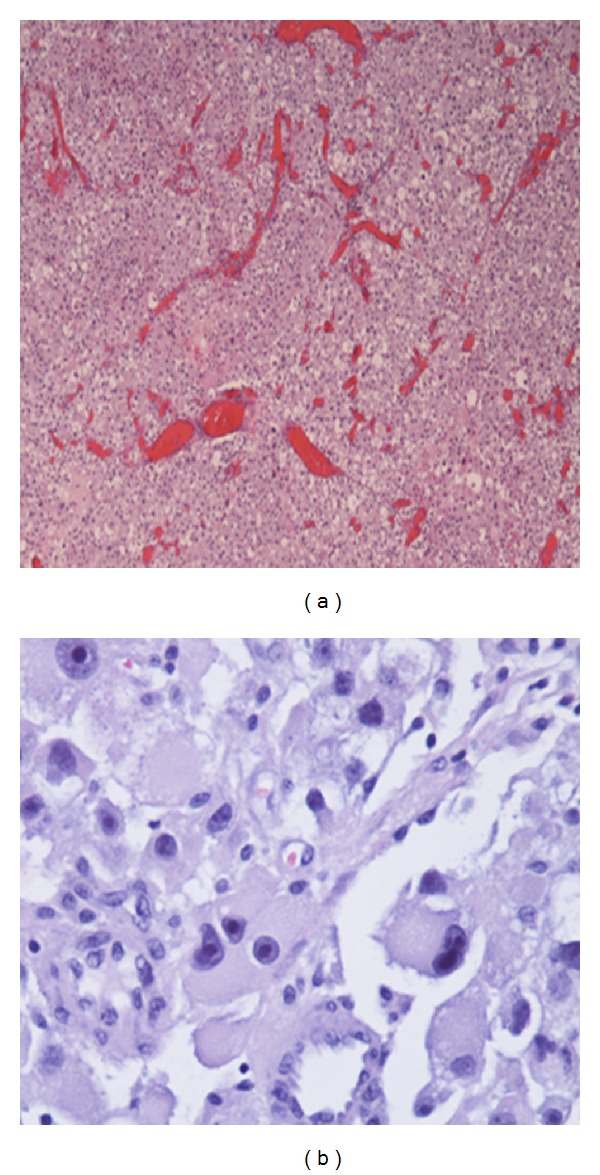
(a) Low power showing an epithelioid tumor with a hemangiopericytoma-like vascular pattern and diffuse pleomorphism. (b) Higher power showing marked nuclear atypia in the form of hyperchromasia, coarse chromatin, prominent nucleoli, and pleomorphism with atypical mitotic figures.

**Figure 5:**
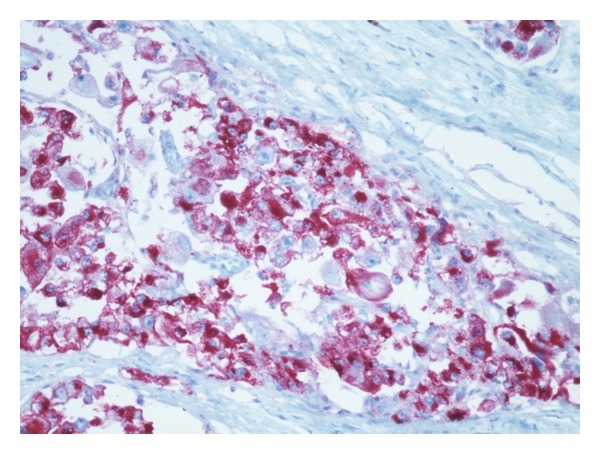
Immunohistochemical stain positive for HMB-45.

The final histopathology results were compatible with a malignant perivascular epithelioid cell neoplasm invading the large and small intestines, with multiple subserosal tumor deposits and extensive tumor studding of the omentum (Figure 4). The immunohistochemical stains were positive for HMB-45, Melan-A, Mart-1, MITF, and cathepsin K, while being negative for S-100, pankeratin, p63, CK5/6, CK8/18, inhibin, chromogranin, synaptophysin, CD117, WT1, myogenin, desmin, SMA, and calretinin (Figure 5). Fluorescent in situ hybridization testing was negative for EWS and TFE3 rearrangement. These findings were most consistent with a malignant perivascular epithelioid cell neoplasm, with the reservation that a S100 negative metastatic melanoma must be excluded clinically. 21 lymph nodes sampled were benign (0/21) and the tumor showed extensive lymphovascular invasion and necrosis. A thorough systemic evaluation ruled out melanoma.

Currently the patient is being treated with everolimus, an m-TOR inhibitor, and has shown good response to this medication.

## 3. Discussion

Perivascular epithelioid cell tumors (PEComas) are rare mesenchymal tumors arising from histologically and immunohistochemically distinctive perivascular epithelioid cells that express both myogenic and melanocytic markers such as HMB 45, HMSA-1, Melan A/Mart 1, microophtalmia transcription factor (Mitf), actin, and less commonly desmin [1, 2]. However, PEComas can modulate their morphology and immunophenotype depending upon microenvironment locations. The term PEComa was introduced by Baek et al. [3]. In 2003 World Health Organization defined PEComas as mesenchymal tumors [4]. They usually occur in young adults, with greater female preponderance [5]. The clinical presentation is usually not specific and a correct preoperative diagnosis is difficult to make, with definitive diagnosis only made after surgery. Preoperative differentials include melanoma, leiomyosarcoma, clear-cell sarcoma, and gastrointestinal stromal tumor [6].

PEComas can be associated with tuberous sclerosis complex, characterized by angiomyolipomas (AML), subependymal giant cell tumors, cutaneous angiofibromas, cardiac rhabdomyomas, lymphangioleiomyomatosis, and pulmonary multifocal micronodular hyperplasia [7, 8].

Malignant PEComas are very aggressive tumors mimicking high-grade sarcomas. Folpe et al. reported 26 cases of PEComas (soft tissue and gynecologic), proposing criteria for the classification of these tumors as benign, of uncertain malignant potential, and malignant. Tumor size >5 cm, infiltrative growth pattern, high nuclear grade, necrosis, and mitotic activity >1/50 HPF were indicators of aggressive behavior [9].

PEComas have been described in different organs and are ubiquitous tumors. In kidneys PEComas represent a wide spectrum of pathology, which include classic AML, microscopic AML, intraglomerular lesions, cystic AML, epithelioid AML, oncocytoma-like AML, and lymphangioleiomyomatosis of the renal sinus [10]. PEComas arising from the bladder and prostate have also been reported [11]. Weinreb et al. reported an unusual case of a PEComa arising from the urachal cyst [12]. PEComas of the lung include clear-cell “sugar tumor” and lymphangioleiomyomatosis (LAM), both of which are positive for HMB-45 [13]. LAM usually affects premenopausal women, characterized by bilateral interstitial proliferation of HMB-45, actin, and desmin positive smooth muscle cells. LAMs in extrapulmonary sites have also been reported [14, 15]. Clear-cell “sugar” tumors are also known to arise in extrapulmonary sites such as the breast, heart, and oral mucosa [16, 17]. Clear-cell myomelanocytic tumor of falciform ligament/ligamentum teres is a PEComa showing predominantly spindle cell morphology [18]. PEComas are also known to involve the female genital tract (vulva, vagina, ovary, and uterus) [17, 19]. PEComas involving the uterus are usually benign, although those associated with tuberous sclerosis may show malignant potential. Fadare et al. described a rare case of uterine PEComa with intra-abdominal tumor studding, involving the ovaries and small intestine. He referred to the above as “PEComatosis” [20].

PEComas can arise in visceral organs such as the pancreas and liver [21, 22]. Pancreatic PEComas are usually discovered incidentally during ultrasound of the abdomen and should be distinguished from clear-cell carcinoma of the pancreas. Both classic and epithelioid AML resembling renal AMLs are known to occur in the liver. Besides kidney and liver, AMLs can occur in nasal cavity, bones, orbit, and pelvis [23, 24]. Although malignant PEComas involving various regions in the gastrointestinal tract such as the jejunum, ileum, cecum, and colon have been reported, PEComas arising in the mesentery are extremely rare [25, 26]. Folpe et al. reported a case of a 46-year-old female with a PEComa arising in the mesentery, with extensive intra-abdominal and hepatic metastases [9]. However, a malignant mesenteric PEComa presenting as an intra-abdominal fistula is a very rare presentation. We did extensive search of literature and to our knowledge this is the first reported case of its kind.

Surgical resection represents the only curative approach for primary PEComa at presentation as well as for local recurrences and distant metastasis, as chemotherapy and radiotherapy have not demonstrated significant benefits [27, 28]. Anthracycline and vincaloid based chemotherapy and/or radiation therapy have been used in the past without obvious improvement in prognosis. Recent studies have shown an overexpression for m-TOR, thus suggesting the role of m-TOR inhibitors in the treatment of these tumors [29]. Rapamycin, an m-TOR inhibitor, has shown significant response in some animal models of tuberous sclerosis [30]. Bergamo et al. reported the use of sirolimus in the management of a large hepatic PEComa [31]. However, more research is needed before these molecular targeted therapies can be widely used for treating these tumors.

Our patient is currently getting treated with everolimus (following surgery), an m-TOR inhibitor, and has been showing good response to this medication.

## 4. Conclusion

PEComas are rare tumors expressing both myogenic and melanocytic markers. Although these tumors have been described in different organs and are ubiquitous, malignant mesenteric PEComa presenting as an intra-abdominal fistula is extremely rare. We did extensive search of literature and to our knowledge this is the first case of its kind in English literature.
